# Estimation of the Production Economic Consequences of Stopping Partial Depopulation in Broiler Production

**DOI:** 10.3390/ani12121521

**Published:** 2022-06-11

**Authors:** Nunzio Sarnino, Anna Catharina Berge, Ilias Chantziaras, Jeroen Dewulf

**Affiliations:** Department of Obstetrics, Reproduction and Herd Health, Faculty of Veterinary Medicine, Ghent University, 9820 Merelbeke, Belgium; cat@bergevetconsulting.com (A.C.B.); ilias.chantziaras@ugent.be (I.C.); jeroen.dewulf@ugent.be (J.D.)

**Keywords:** poultry, production, thinning, biosecurity, *Campylobacter*, partial depopulation

## Abstract

**Simple Summary:**

Partial depopulation is often used in broiler production to optimize the use of the farm space and rear a larger number of broilers. However, it may increase the risk for the introduction of *Campylobacter* spp. in the poultry house. A simulation was performed to evaluate the production consequences of a Belgian poultry house performing a 25% partial depopulation at 35 days of age compared with a scenario where the entire flock is slaughtered at 42 days of age. The result showed that stopping partial depopulation leads to a substantial production and profit decrease. To compensate the loss, it would be necessary an increase in meat price.

**Abstract:**

Partial depopulation is the removal and slaughter of part of a flock prior to the final slaughter age, and this practice allows broiler producers to optimize stocking density in broiler houses. However, this practice constitutes a serious break in farm biosecurity that can lead to the introduction of various pathogens in the flock, including *Campylobacter* spp. In this study, the production of a house performing partial depopulation of 25% of the flock at 35 days of age prior to the final slaughter at 42 days was compared with a production system where partial depopulation was not performed. The differences in production costs, profit, and technical performance parameters were evaluated. The model indicated that stopping partial depopulation reduces the production between 16 to 24%, which results in a 14% reduced profit per kg of live weight, and a 31% reduced profit per production cycle. To compensate the profit loss, it would be necessary to increase the meat price 3% from a starting price of 87.44 cents. For current conventional broiler production, it may be financially challenging to stop partial depopulation practices. Focusing on external biosecurity to avoid the introduction of *Campylobacter* into poultry houses may be the right compromise.

## 1. Introduction

Partial depopulation in broiler production consists of the removal and early slaughter of a portion of the flock prior to final slaughter age. Partial depopulation is used at different ages and different percentages in various countries. In Belgian conventional production, approximately 25% of the flock are slaughtered at 35 days, and the remaining flock at 42 days of age [1]. From an economic point of view, partial depopulation allows producers to have more efficient use of the predetermined floor space with a higher number of animals per square meter [2], while still respecting the EU regulations that set the maximum stocking density in a house at 42 kg/m^2^ [3]. Stopping partial depopulation practices may result in a lower number of one-day-old chicks placed, and subsequently a lower number of animals sent to slaughter [4]. While performing partial depopulation, the maximum density allowed by law is reached faster, and a part of the flock is sent for slaughter earlier while the remaining animals continue until the end of the production round. Several studies have found association between partial depopulation and the introduction of *Campylobacter* spp. in poultry houses [5,6,7]. 

Campylobacteriosis is one of the most common foodborne gastrointestinal diseases in the European Union (EU), with more than 200,000 cases reported every year; the actual number of cases has been estimated at nine million [8]. In Belgium, the financial burden of campylobacteriosis was estimated at 10.9 million € in 2007 [9]. Partial depopulation may be a risk for the introduction of *Campylobacter* for various reasons, such as a break in the farm biosecurity due to the early catching of birds [10], or as stress in the remaining flock after the partial depopulation [11]. Partial depopulation is a stressful event for the broilers that are deprived of food and water several hours before the arrival of the catching crew [12]. The catching crew may introduce *Campylobacter* into the flock, as the bacteria have been found on boots, clothing, and other equipment prior to being used during the partial depopulation [5]. Furthermore, broiler transport crates are often contaminated with *Campylobacter* [13,14,15].

It has been estimated that stopping partial depopulation may not be the best cost-effective intervention to reduce *Campylobacter* prevalence in poultry houses, especially compared with other biosecurity interventions [16,17]. In any case, there are no studies that have quantified the economic consequences of stopping partial depopulation for a poultry farm. 

The goal of this study is to estimate production and economic impacts for Belgian broiler producers in a scenario where 25% partial depopulation is not performed, taking into consideration technical performance parameters. Furthermore, possible solutions to compensate for the eventual economic losses incurred when no partial depopulation is performed are explored.

## 2. Materials and Methods

A stochastic simulation was performed to estimate the live weight broiler production, costs, and profits of a Belgian poultry house in a 25% partial depopulation scenario at day 35, prior to final depopulation at day 42, and a no partial depopulation scenario where all birds are slaughtered at day 42. 

Data for costs and income, as well as production parameters used in the first step of building the model (Table 1), were from the Flemish Government Market Information [1]. The farm size data was from 2018. For costs and income, the data distributions from 2009–2018 were used, and for day-old chicks, distribution from 2013–2018 were used. The complete list of costs and incomes is included in the Supplementary Materials (Table S1). All birds were Ross 308. The costs and income data used were for kg of live weight produced, except day-old chicks (€ cents per bird) and fixed costs (€ per m^2^). Distributions for mortality rate, cycles per year, percentage of partial depopulation, and the weight of the broilers at partial and final depopulation were calculated from the mean of the production window 2009–2018. For costs and income distributions, normal or triangle distributions were used, and for each parameter, the best fitting between these two distributions was selected.

The maximum stocking density for the broiler house was based on EU legislation allowing 42 kg/m^2^ under special circumstances [3]. Broiler weights and growth rates were based on the ROSS 308 mixed gender (fast feathering) 2019 performance index [18]. Total production costs were calculated by summing all single production costs. The model was developed using Excel (Office 2016) for the deterministic part and @RISK (Industrial Version 8.1.1) for the distributions, the stochastics simulation, and sensitivity analysis. The stochastics simulation was performed with 100,000 iterations.

### 2.1. Difference in Final Weight between 25% Partial Depopulation and No Partial Depopulation Production Due to Different Stocking Density

The stocking density is the broiler weight per square meter. As stocking density can be a parameter that influences the final weight, it was taken into consideration to estimate the total kg of live weight produced. The maximum stocking density used in this simulation was 42 kg/m^2^. In this simulation, the assumptions of Kryeziu (2018) [19], comparing rearing flocks at different stocking densities–high (22 broilers/m^2^) and low (14 broilers/m^2^)–were used to estimate the difference in final weight. The study estimated an increase of 5.6% (SD 1.2%) in final weight at 42 days when switching from high to low stocking density, weighing the birds at 35 and 42 days. The number of broilers per square meter in this simulation was similar to those used by Kryeziu, with 21 broilers/m^2^ at 25% with partial depopulation, and 15 broilers/m^2^ when not performing partial depopulation. 

The maximum number of broilers reared at 25% partial depopulation (Mp) was calculated as:

Mp= D ÷ Wf × Fs ÷ %pd

While, in case of no partial depopulation, the maximum number of broilers (Mnop) was calculated as:

Mnop= D ÷ Wf2× Fs

where:

D = maximum density (42 kg/m^2^)

Fs = farm size

%pd = percentage of the flock partially depopulated

Wf = weight of broilers slaughtered at final depopulation at 25% partial depopulation (kg)

Wf2 = weight of broilers slaughtered at final depopulation without partial depopulation (kg)

The kilograms of live weight produced in a production cycle were calculated as the number of broilers per production cycle minus the mortality rate, multiplied by the weight of the broiler at partial or final depopulation.

For the 25% partial depopulation scenario, kilograms of live weight produced per cycle (Kgpd) are calculated as:

Kgpd=((Bp × Wp)+(Bf × Wf)− M)

In the case of not performing the partial depopulation, kilograms of live weight produced per cycle (Kgnopd) are calculated as:

Kgnopd=((Bfd × Wf2)− M) 

where:

Bp: Number of broilers slaughtered at partial depopulation 

Wp: Weight of broilers slaughtered at partial depopulation (kg)

Bf: Number of broilers slaughtered at final depopulation 

Wf: Weight of broilers slaughtered at final depopulation (kg)

Bfd: Number of broilers slaughtered at depopulation; no partial depopulation scenario

Wf2: Weight of broilers slaughtered at depopulation; no partial depopulation scenario (kg)

M: Mortality rate

### 2.2. Feed Costs

Feed represents up to 70% of broilers’ production costs. To calculate the feed cost per kg of live weight, the performance data of Ross 308 Performance sheet [18] and the average feed cost in Belgium (331.57 €/ton, SD 29.66 in 2013–2018) [1] were used. No estimated difference in final weight was taken into consideration to estimate the difference in feed costs.

The feed price per kg of live weight in the case of not performing partial depopulation was calculated as:

F42= Ci42× Fp ÷ Bw42

where: 

F42 = feed price per kg of live weight produced; slaughtering broilers at 42 days (€/kg)

Ci42 = broiler cumulative intake at 42 days (kg)

Fp = feed price (€ per kg of feed)

Bw42 = broiler body weight at 42 days (kg)

To calculate the feed price per kg of live weight in case of 25% partial depopulation, it was necessary to first calculate the feed price per kg of live weight at 35 days, as follow:

F35= Ci35× Fp / Bw35

where:

F35 = feed price per kg of live weight produced; slaughtering broilers at 35 days (€)

Ci42 = broiler cumulative intake at 3 days (kg)

Fp = feed price (€ per kg of feed)

Bw35 = broiler body weight at 35 days (kg)

For partial depopulation scenario, the cost of feed (Fc) per kg of live weight was calculated as:

Fc =(F35× % pd))+(F42×(1− % pd)

where: 

F35 = feed price per kg of live weight produced; slaughtering broilers at 35 days (€)

F42 = Feed price per kg of live weight produced; slaughtering broilers at 42 days (€/kg)

%pd = percentage of the flock partially depopulated

### 2.3. Day-Old Chicks Costs

The price per kg of live weight of day-old chicks (P1) was calculated as:

P1=Bp × Kg ÷ Nb

where: 

Bp = price of one day-old chick (€)

Kg = kg of live weight produced in a production cycle 

Nb = number of broilers per cycle

### 2.4. Other Variable Costs

Due to insufficient accessible production data regarding the difference in variable costs at different flock densities, it was assumed that the costs per kg of live weight were the same in the two scenarios. Water consumption was not taken into consideration due to lack of data. 

### 2.5. Fixed Costs per Cycle

Fixed costs represented the costs for ground and structures, tools, depreciation, interests, and other fixed costs. The estimation of fixed costs was based on €/m^2^ since in this simulation, the use of existing buildings and equipment was assumed to switch from 25% partial depopulation to no partial depopulation. 

Fixed costs per kg of live weight (Fxc) were calculated as follows:

Fxc=((Cm × Fs) )/((Kg × Cb))

where:

Cm = fixed costs per m^2^

Fs = farm size (m^2^)

Kg = kilograms of live weight produced in a production cycle

Cb = number of production cycles per year

### 2.6. Profit

The gross income was mostly from the live weight of chickens sold. A small income was also derived from changes in livestock value, and this change of livestock value depends on market variation and can also be negative [1].

The meat revenue price was 87.44 cents/kg (SD 4.70); income from the change in livestock value was 0.64 cents/kg of live weight (SD 0.98). 

The focus was on the profit per kg of live weight and for the profit per production cycle.

The profit per kg of live weight produced was calculated as follows:

 Pk =(Rev + L)− Ct

where:

Pk = profit per kg of live weight produced

Rev = revenue price per kg of live weight produced

L = change in livestock value

Ct = total production costs

The profit per production cycle (Ppc) was calculated as follows:

Ppc=Pk × Kg

where:

Pk = profit per kg of live weight produced (€)

Kg = kilograms of live weight produced per cycle

### 2.7. Price Break Even

The price breakeven (Pbe) is the price that a producer would need to sell the birds to compensate for the economic loss of switching from 25% partial depopulation to no partial depopulation.

It was calculated as:

Pbe=( P25+Kgnop ×Cnop)/Kgnop

where:

P25 = profit per cycle (€)

Kgnop = kilograms of live weight per cycle produced in no partial depopulation scenario

Cnop = production cost when not performing partial depopulation (€)

## 3. Results

### 3.1. Costs

The model indicates that total production cost in the no partial depopulation scenario is 0.95% higher than in 25% partial depopulation, with a maximum of 1.97% (SD 0.24%) (Table 2).

Average fixed costs are higher with no partial depopulation (5.82 cents/kg of live weight, SD 0.37) compared to 25% partial depopulation (4.66 cents/kg of live weight, SD 0.30).

In the 25% partial depopulation scenario, the average feed cost is 54.61 cents per kg of live weight (SD 4.07), while in the no partial depopulation scenario the average feed cost is 55.79 cents/kg of live weight (SD 4.16). Thus, in the no partial depopulation scenario, mean feed price is 2.16% (SD 0.08) higher.

The average day-old chicks’ cost per kg of live weight is lower in the no partial depopulation scenario (12.83 cents, SD 0.57) than at 25% partial depopulation (13.83 cents, SD 0.57).

### 3.2. Farm Production and Profit

The estimation of the difference in the production of kg of live weight is 20% (min. 16%, max. 24%, SD 0.79%), switching from the 25% partial depopulation scenario to the no partial depopulation scenario (Table 3).

The average profit per kg of live weight at 25% partial depopulation is 5.34 cents, while at no partial depopulation the profit per kg of live weight decreases to 4.55 cents, with an economic loss of 14%.

In this model, the maximum number of broilers per production cycle when performing partial depopulation was 81,312, while the number dropped to 57,757 when stopping partial depopulation. The average difference in profit per cycle is estimated to be 31%, with an average profit of 10,199 € per cycle at 25% with partial depopulation and 6958 € without partial depopulation. 

### 3.3. Price Breakeven

The estimated average revenue price breakeven (Table 4) is 90.20 cents with an average difference between original meat revenue and price breakeven of 2.76 cents (difference 3%, 90% CI ± 0.01%).

## 4. Discussion

This simulation study indicates that that ceasing partial depopulation practices of broiler production in the Belgian broiler market may reduce the kg of live weight of broilers produced up to 20%. This will decrease the profit per kg of live weight produced by approximately 14%. Considering that stopping partial depopulation reduces the amount of meat produced, the loss in profit per cycle is approx. 30%.

The higher production costs strongly affect the profit decrease of the no partial depopulation scenario. Feed cost per kg of live weight is the parameter that most increases the total production costs in a no partial depopulation system. This parameter is positively influenced by the percentage of partial depopulation performed since the feed conversion ratio in broiler is lowest in the early life of the birds and becomes higher with increasing bird age. Slaughtering a part of the flock one week or more before the final slaughter age leads to a lower average feed conversion ratio, and thus to lower feed cost per kg of live weight. In Belgium, where 25% of the flock is commonly slaughtered at 35 days and the remaining birds at 42 days, stopping partial depopulation may lead to an increase in the feed price of about 2%. In countries where a higher percentage of the flock is partially depopulated, or where the partial depopulation can occur multiple times, for example, the UK, the difference in feed cost per kg of live weight switching from partial depopulation to no partial depopulation may be even higher. This would happen because the average feed conversion ratio of the flock would be higher.

The other parameters that negatively influence the total production costs of the no partial depopulation scenario are the fixed costs per kg of live weight. Fixed costs include the purchase of tools, ground, structures, the interests, and the depreciations. In this simulation, it was hypothesized that after stopping partial depopulation, the producer would continue to use the existing production infrastructures without any modification. Thus, with the existing production infrastructure, the no partial depopulation scenario leads to a lower number of broilers per production cycle produced and this means that the fixed costs are spread between a lower number of birds.

On the other hand, the price of day-old chicks per kg of live weight produced is lower in systems without partial depopulation. The reason is that with a single bird (price per chick 32.11 cents, SD 1.02), more kg of live weight is produced. Thus, with the increasing percentage of the flock that is slaughtered earlier, the day-old chicks cost per kg of live weight increases.

The reduction in average stocking density in case of stopping partial depopulation is a parameter that may positively influence broilers’ final weight. Producing broilers without partial depopulation means that the initial number of animals is lower compared with a scenario where one, or more, partial depopulations are planned. In this simulation, with 25% of the flock partially depopulated, the average number of broilers per m^2^ was 21, while in case of stopping partial depopulation the number decreased to 15 per m^2^. The increase in final body weight at final slaughter in case of stopping partial depopulation could be explained by the increase in feed intake rearing broilers at lower densities [20,21]. 

One of the largest productivity challenges for producers of the no partial depopulation production is the sub-optimal use of the house space, and this means a lower profit per production house due to the lower mean stocking density. Due to the EU regulations on maximal stocking density that entered into force in 2007, producers have been developing systems to increase stocking density throughout the production cycle using partial depopulation. According to Council Directive 2007/43/EC [3], under certain circumstances of low mortality and well-experienced producers, stocking density can reach 42 kg/m^2^, and this is the common stocking density of large poultry farms in Belgium. Stopping partial depopulation and therefore reducing the stocking density is not an attractive option for the current fast-growing broiler production. In the UK, the Royal Society for the Prevention of Cruelty to Animals (RSPCA) banned the practice of partial depopulation [22], then retracted their decree and instead created a derogation for producers to continue to use partial depopulation because the ban was deemed unaffordable for producers and consumers.

A decrease in profit of around 30% or more would naturally not encourage producers to stop partial depopulation. Even in Sweden, where complete final depopulation at 35 days has been the standard system, some major poultry producers are interested in using more partial depopulation systems to maximize their profit [23].

On the other hand, the increase in stocking density may negatively affect the health of the broilers due to the increase in cellulitis, hock burns, scratches, and foot-pad dermatitis [24,25]. However, Alfifi (2020) [26] declared that thinning may have a protective effect on dermatitis, allowing the remaining broilers to have more space in the final production phase. Differences in health costs, labour costs, electricity use, and manure disposal between flocks at different densities were not included in the model, due to lack of data. The mortality rate is most likely marginally influenced by stocking density, since it appears that mortality is more influenced by genetic differences, quality of chicks, and management [19,21,27]. Poultry experts have indicated that health costs may be lower and management easier in no partial depopulation production whereas labour costs may not be impacted, however, there are limited data supporting these opinions [23,28].

A limitation of the model was that it was developed using a single data set, the most recent data being from 2018. Therefore, the results do not strictly apply to other production systems with different percentages of partial depopulation but may be a good indication in countries with a similar production system and similar percentage of flock being partially depopulated. Unfortunately, there were a lack of data on technical performance parameters at different densities, such as electricity or water consumption. In further studies, it would be interesting to expand the model using wider data from different datasets and different countries, as well as the inclusion of a sensitivity analysis to evaluate the effect of the various parameters on the results. Another limitation of the study was the limited number of observations regarding Belgian broiler production. To determine the values used in this simulation, a distribution of data from the period 2009–2018 were used. Unfortunately, only one observation per year was available.

There are discordant opinions regarding the effect of partial depopulation in the introduction and prevalence of *Campylobacter* in poultry houses. Hertogs (2021) [7] found an increase in prevalence of 55% after partial depopulation, most likely due to contamination to harvesting equipment and materials used by the catching crew. Koolman (2014) [2] identified partial depopulation as a major risk for the introduction of *Campylobacter* in poultry farms, as all the *Campylobacter*-negative farms participating in the study became positive after partial depopulation. However, a study of 1,737 flocks at two Dutch integrators found no increased risk of *Campylobacter* introduction due to partial depopulation [15]. Ellis-Iversen (2012) [29] considered the age of the broilers a more important risk factor. 

In a report by the ICF GHK consulting service, stopping partial depopulation was one of the possible interventions considered, but it had both lower efficiency (10–25% prevalence reduction) and higher cost per campylobacteriosis DALY avoided compared to other interventions such as improved biosecurity or using hot water on the carcasses. For this reason, stopping partial depopulation was not selected as an optimal intervention [16].

In Van Wagenberg (2016) [17], the cost-effectiveness of interventions to reduce campylobacteriosis in six European countries indicated that stopping partial depopulation had one of the highest cost-effectiveness ratios and leads to a lower prevalence reduction in poultry compared with other interventions such as applying separate designated tools in each house and building an anteroom with hygiene barrier in each house. Furthermore, it estimated a modest prevalence decrease of 3.9% of *Campylobacter* in poultry farms after stopping partial depopulation. In the Netherlands, with an initial flock prevalence of 24.2%, the estimated prevalence was 21.1% without partial depopulation. Despite a similar prevalence reduction with other selected interventions, as for example the use of an anteroom with hygiene barrier, Van Wagenberg estimated a cost of stopping thinning in the Netherlands of 158,213 € per avoided DALY, while the estimated cost of anteroom with hygiene barrier was 189€ per avoided DALY. Even in countries with high numbers of *Campylobacter*-positive flocks, such as Spain or Poland, the estimated reductions are between 3.8 and 5.9% without partial depopulation.

Allen (2008) [5] provided evidence of the connection between partial depopulation and the introduction of *Campylobacter* in poultry houses, addressing the contamination to the catching crew. Catchers still perform partial depopulation in *Campylobacter*-positive flocks, and it is likely that the pathogens stay on vehicles, equipment, and members of the crew. For this reason, the focus to avoid the introduction of *Campylobacter* should be on biosecurity measures, such as more frequent changing of protective clothing by the catching team, an increased number of hygiene barriers, disinfectant footbaths, and a more thorough disinfection of vehicles associated with the partial depopulation procedure. The importance of biosecurity during partial depopulation is exacerbate by the fact that after partial depopulation it is not possible to clean and disinfect the poultry house, and once the pathogens enter the house, they rapidly spread among the remaining birds [30]. Van Limbergen (2018) [31] performed a study on the biosecurity on 399 broilers farms using the Biocheck tool [32], and identified the entrance of visitors and staff as the category with the lowest biosecurity score (thus high biosecurity risk) and one of the critical points where the farm biosecurity could improve. 

From a survey conducted with 150 British poultry producers, it emerged that stopping partial depopulation was the least attractive between 11 biosecurity interventions to reduce *Campylobacter* prevalence in broiler houses, and the one considered less economically feasible by the producers. Other interventions such as providing specific overall and boots for each house were considered both easier to be applied and more economically feasible. Even between-batch biosecurity measures, such as increasing the downtime between batches, were deemed more attractive than stopping partial depopulation [33]. The risk of introducing *Campylobacter* during partial depopulation may be reduced using a platform at the entrance of the poultry house, where members of the catching crew must clean and disinfect boots and shoes, as well as machines and crates [26]. The general belief is that instead of stopping partial depopulation, more effort should be put towards training the catchers and the staff of the poultry houses, and in general to improve the farm external biosecurity to reduce the risk that *Campylobacter*-negative flocks become contaminated.

Since poultry and human campylobacteriosis are strictly related [34], reducing the prevalence of *Campylobacter* in broiler farms can bring a reduction in campylobacteriosis burden for the public health system. For this reason, a financial support system based on the reduction in *Campylobacter* prevalence in poultry houses may be a good compromise both for governments and poultry producers.

A study in the UK showed how financial incentives for commercial broiler farms may cause a reduction in the prevalence of *Campylobacter*. The producers that received an incentive had a reduction of 54% in the odds of their houses being highly contaminated with *Campylobacter* [35]. Besides the break in house biosecurity, partial depopulation is also an animal welfare issue. Prior to partial depopulation, the feed is withdrawn for up to 8 hours, followed by a water deprivation for up to one hour, an event that stresses the remaining flock [5]. If in the future the choice is to stop the partial depopulation practice, an increase in meat price will be inevitable to compensate the production reduction an increased production costs.

Modern consumers are increasingly interested in animal welfare. In the Netherlands, a kind of broiler production known as “beter leven” (better life) with stricter rules in terms of density (broilers/m^2^), alimentation, and house conditions than regular broiler production has been implemented [36]. The farms rearing under this label, despite having higher production costs, have a higher income compared with conventional ones [37]. The cost of the meat is higher, but some consumers are willing to pay more for meat from a production system with a higher focus on animal welfare [38]. In Belgium, the trend in poultry meat consumption is also changing and several big supermarkets have announced that they will move towards only selling meat from slow-growing breeds with stricter rules regarding housing, age at slaughter, and stocking density [39]. It is not clear how this trend will affect partial depopulation. If the regulations regarding animal welfare continue to allow partial depopulation, it may be economically convenient for producers to continue with this practice, to rear the maximum amount possible of broilers, and optimize the use of the house space. If the producers decide to stop partial depopulation, then an increase in meat price and eventually subsidies for such farms may be a possible solution to keep broiler production economically sustainable.

## 5. Conclusions

Stopping partial depopulation, which might reduce *Campylobacter* contamination of birds slaughtered, can be a great challenge for current Belgian broiler producers. It can lead to a substantial production decrease due to the sub-optimal use of existing structures. Feed costs per kg of live weight produced may be higher due to the increase in the average age of the flock, as well as fixed costs per kg of live weight, leading to higher total production costs. The cost per kg of live weight of day-old chicks may be positively affected by stopping partial depopulation. The profit loss per production cycle, switching from 25% partial depopulation to no partial depopulation, was estimated to be about 30%. To compensate the economic loss, the most straight-forward solution would be an increase in price per kg, estimated to be approximately 3%.

## Figures and Tables

**Table 1:** Production parameters used in the first step of building the model.

Input Data	Estimate	SD *
House floor space holding birds (m^2^)	3764	
Max density (kg/m^2^)	42	
Cycles per year	6.91	0.14
Mortality (%)	3.43	0.20
% flock for partial depopulation	25%	0.55
Weight (kg) broilers at 35 days (25% partial depopulation)	1.94	0.04
Weight (kg) broilers at 42 days (25% partial depopulation)	2.59	0.08
Weight (kg) broilers at 42 days (no partial depopulation)	2.74	0.09

* SD = standard deviation.

**Table 2:** Production costs per kg of live weight produced in the 25% partial depopulation scenario and no partial depopulation scenario.

Production Costs per kg of Live Weight (Cents)		
Outputs	25% Partial Depopulation	No Partial Depopulation
Average	82.73	83.52
Maximum	96.75	97.70
Minimum	73.85	74.33
Standard deviation	4.19	4.29
90% confidence interval	±0.02	±0.02

**Table 3:** Production and profit results in 25% partial depopulation scenario and no partial depopulation scenario.

Outputs	25% Partial Depopulation	No Partial Depopulation
Kg of live weight/cycle		
Average	190,756	152,661
Maximum	200,341	154,141
Minimum	182,769	151,079
Standard deviation	1947	328
90% CI	±1013	±1.71
Average	190,756	152,661
Profit/kg of live weight (cents)		
Average	5.34	4.55
Maximum	31.03	30.42
Minimum	20.46	21.54
Standard deviation	6.39	6.45
90% CI	±0.03	±0.03
Profit per cycle (€)		
Average	10,199	6958
Maximum	59,815	46,505
Minimum	−39,354	−33,029
Standard deviation	12,193	9855
90% CI	±63	±51
Average	10,199	6958

**Table 4:** Price breakeven.

Price Breakeven (Cents)	
Outputs	
Average	90.20
Maximum	116.35
Minimum	62.23

## Data Availability

All data used in the analysis are provided and appear in the submitted article.

## Supplementary Materials

The following supporting information can be downloaded at: https://www.mdpi.com/article/10.3390/ani12121521/s1, Table S1: Complete list of costs and incomes.
